# A phase II clinical trial of a Vi-DT typhoid conjugate vaccine in healthy Indonesian adolescents and adults: one-month evaluation of safety and immunogenicity

**DOI:** 10.1186/s40794-023-00210-z

**Published:** 2024-02-01

**Authors:** Sukamto Koesnoe, Bernie Endyarni Medise, Iris Rengganis, Sri Rezeki Hadinegoro, Mita Puspita, Rini Mulia Sari, Jae Seung Yang, Sushant Sahastrabuddhe, Soedjatmiko, Hartono Gunardi, Rini Sekartini, Angga Wirahmadi, Aria Kekalih, Sreshta Mukhi, Hindra Irawan Satari, Novilia Sjafri Bachtiar

**Affiliations:** 1 Department of Internal Medicine, Faculty of Medicine Universitas Indonesia, Dr. Cipto Mangunkusumo General National Hospital, Jakarta, Indonesia; 2 Department of Child Health, Faculty of Medicine Universitas Indonesia, Dr. Cipto Mangunkusumo General National Hospital, Jalan Diponegoro no 71, Jakarta, 10340 Indonesia; 3 Community Medicine Department, Faculty of Medicine Universitas Indonesia, Dr. Cipto Mangunkusumo General National Hospital, Jakarta, Indonesia; 4 PT. Bio Farma, Bandung, Indonesia; 5 International Vaccine Institute, Seoul, Republic of Korea

**Keywords:** Typhoid Fever, Typhoid conjugate vaccine, Vi-DT, Vi-PS

## Abstract

**Background:**

Typhoid fever is commonly found until today, especially in developing countries. It has fatal complications and measures must be taken to reduce the incidence of typhoid. Vaccinations are a key factor in prevention. This is a phase II randomized observer-blind clinical trial on a novel Vi-DT conjugate vaccine on 200 subjects 12 to 40 years of age.

**Methods:**

Subjects were screened for eligibility after which a blood sample was taken and one dose of vaccine was administered. Investigational vaccine used was Vi-DT and control was Vi-PS. Twenty-eight days after vaccination, subjects visited for providing blood sample to assess immunogenicity and were asked about local and systemic adverse reactions that occurred in the first 28 days.

**Results:**

Subjects had minor adverse reactions. Pain was the most common local reaction. Muscle pain was the most common systemic reaction. There were no serious adverse events up to 28 days post vaccination. Seroconversion rates were 100% in the Vi-DT group and 95.96% in the Vi-PS group. Post vaccination GMTs were increased in both groups but it was significantly higher in the Vi-DT group (p < 0.001).

**Conclusions:**

Vi-DT typhoid conjugate vaccine is safe and immunogenic in healthy Indonesian subjects 12 to 40 years.

**Trial registration:**

Approved by ClinicalTrials.gov. Clinical trial registration number: NCT03460405. Registered on 09/03/2018. URL: https://clinicaltrials.gov/ct2/show/NCT03460405.

## Introduction

Typhoid fever and paratyphoid fever, also known as enteric fever, is a multisystem disease that imposes a significant public health problem especially in developing countries [1]. The mode of transmission is fecal-oral and it is most common in overcrowded areas with poor sanitation and hygiene [2–4]. In high income countries, typhoid is the most common vaccine preventable disease found in international travelers coming from countries with a high incidence of the disease [5].

Globally, about 215 000 deaths occur from about 26 million cases of typhoid and paratyphoid fever per year [6, 7] Data from CDC showed that in 2019 in South-East Asia, there were 306 cases of typhoid fever per 100,000 persons [8, 9].

Typhoid fever has severe complications such as intestinal perforation and gastrointestinal hemorrhage that often end fatally [1, 10]. With appropriate treatment and antimicrobials, the case fatality rate (CFR) was 1 to 4% whereas without treatment, the CFR was as high as 10 to 20% [11]. Measures must be taken to reduce the incidence of typhoid fever to minimize mortality from this illness. Although improving sanitation and hygiene as well as availability of clean water and food are important for prevention of typhoid fever, vaccination is an important tool to reduce overall incidence [2, 12].

The World Health Organization has recommended the use of typhoid vaccines since 2008, however many countries have not included it in their routine immunization program [13] because Vi-polysaccharide vaccines are poorly immunogenic [14, 15] and not licensed for children below 2 years of age as well as have a limited duration of immunity and hence required repeated booster doses. These limitations could be minimized by conjugating the polysaccharide to a carrier protein thereby creating a typhoid conjugate vaccine. TCVs have been proven to be safe, immunogenic and do not have the shortcomings of the previously licensed vaccines. An example of a typhoid conjugate vaccine that has been licensed by WHO is Typbar TCV, manufactured by Bharat Biotech and has been proven to be safe and immunogenic through phase I- III trials [14–16].

This is a phase II clinical trial of a novel Vi-DT typhoid conjugate vaccine (TCV) formulated by BioFarma and International Vaccine Institute and aimed to evaluate safety and immunogenicity in subjects 6 months to 40 years. Vi-DT involves a conjugation between Vi and diphtheria toxoid. The results of the phase I trial and phase II trial of Vi-DT vaccine on infants and children were found to be satisfactory and have already been published [17–19].

## Methods

### Study design

This trial used a randomized observer-blind design with 200 subjects 12 to 40 years of age. One hundred subjects were administered with an investigational vaccine (Vi-DT TCV) whereas the other hundred were administered with Vi-polysaccharide (Vi-PS) as control.

### Sample size

The study required 82 subjects in each group to reject the null hypothesis that seroconversion of the two groups is equal with probability power of 0.9. Assuming a 20% dropout rate, 100 subjects were enrolled in each group.

### Study procedures

Subjects were given detailed information on the vaccine, solicited and unsolicited adverse events, visit schedule and contact person details. If they agreed to abide by the rules of the trial, they were given the informed consent form. After signing the informed consent form, inclusion and exclusion criteria were evaluated.

Healthy subjects who were willing to follow the rules of the trial as well as follow-up schedule and willing to sign an informed consent form were included in the trial.

Exclusion criteria were subjects who were enrolled in another trial, had a fever, had a history of allergy to the vaccine component, had a coagulopathy history, were undergoing treatment likely to alter immune response, had a chronic disease and had a history of typhoid fever. Subjects who previously received any typhoid vaccination or any other vaccine 1 month prior to vaccination and subjects who planned to leave the study area before trial completion were also excluded.

Both the trial and control vaccines were given codes, known only to the unblinded team involved in vaccine administration. The sequence of these codes was generated using a randomization website. After evaluation of inclusion and exclusion criteria by the blinded team of investigators, subjects were vaccinated with either Vi-DT or Vi-PS by the unblinded team of vaccinators based on the randomized sequence.

A blood sample was obtained prior to vaccination. Twenty-eight days post vaccination subjects were called for blood sampling and to report any adverse local and systemic reactions that occurred since vaccination. This trial was conducted in 2 primary health centers in Jakarta, Indonesia namely Senen primary health center, located in Central Jakarta and Jatinegara primary health center, located in East Jakarta.

### Investigational product and control

Vi-DT vaccine manufactured by BioFarma was used as an investigational product. Each dose of this vaccine (0.5 mL) is composed of 25 µg of a purified Vi capsular polysaccharide of S. Typhi, 5 mg of 2-phenoxyethanol as preservative and 0.5 mL of phosphate buffer solution. IP was stored at a temperature of + 2 °C to + 8 °C and is in the form of multi-dose vials. Licensed Vi-PS vaccine (Typhim Vi® produced by Sanofi) was used as a control. Control was also stored at a temperature of + 2 °C to + 8 °C but is in the form of pre-filled syringes. Both vaccines were administered intramuscularly in the deltoid region.

### Safety and immunogenicity evaluation

Subjects were monitored up to 30 min post vaccination to evaluate immediate local and systemic adverse reactions. Subjects were given diary cards where they had to record their daily temperature and note down any delayed local and systemic reactions that occurred after 30 min up to 28 days post vaccination. Reactions that were monitored were solicited reactions such as pain, redness, swelling, induration, fever, muscle pain, fatigue and unsolicited reactions such as vomiting, diarrhea etc. Subjects also had to record the severity of the reactions and how long they lasted. Subjects were given a telephone number of a contact person they could get in touch with if they experienced any worrisome adverse effects, had any enquiries or experienced serious adverse events. Causality of adverse events to the vaccines were determined by a data safety monitoring board (DSMB).

For immunogenicity of Vi-DT, anti-Vi IgG titers in sera were assessed pre-vaccination and 28 days post-vaccination. The ELISA method recommended by the WHO Expert committee on Biological standardization [20] was used. Geometric mean titer (GMT) and seroconversion were calculated. Seroconversion was defined as percentage of subjects with antibody increment ≥ 4 times compared to baseline.

Data for safety and immunogenicity were analyzed using SPSS.

## Results

**Fig. 1:**
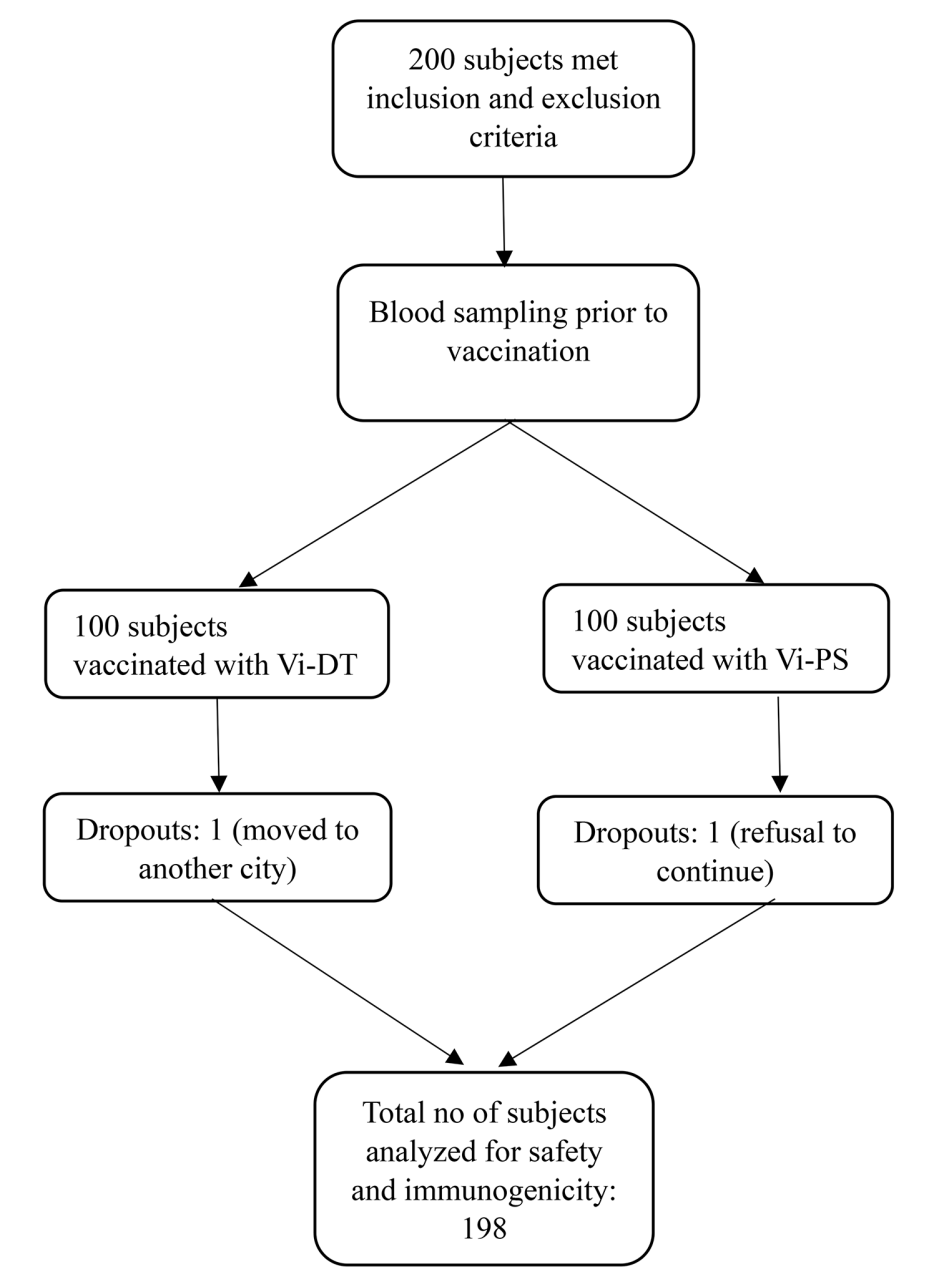
Study flowchart

Two hundred subjects of 12–40 years of age were recruited and followed up from July 2018 up to January 2019 (See Fig. 1). Table 1 shows the demographic data of the subjects. At the end of 28 days there were 2 dropouts, 1 in the Vi-DT group and 1 in the control group, hence analysis was done on 198 subjects. Reason for dropout in the Vi-DT group was because the subject moved to a different city and could not be contacted. Reason for dropout in the Vi-PS group was because the subject refused to continue the trial.

### Immunogenicity

Table 1 shows immunogenicity of Vi-DT compared to Vi-PS. Anti-Vi IgG titer was measured 28 days post vaccination and compared to the baseline. Seroconversion rates were 100% for Vi-DT and 95.96% for Vi-PS. Although the seroconversion rate was higher in the Vi-DT group, it was insignificant compared to Vi-PS. Geometric Mean Titer (GMT) of antibody following vaccination. The post vaccination GMT was significantly higher in Vi-DT compared to Vi-PS (p < 0.001).

**Table 1:** Demographic data and immunogenicity

	Vi-DT	Vi-PS
Gender		
Males (%)	45	40
Females (%)	55	60
Mean age (years)	25.26	25.18
Seroconversion ≥ 4 times (%) at 28 days compared to baseline	100%	95.96%
Pre-vaccination GMT (IU/mL)	0.0226103	0.0048849
28 days post-vaccination GMT (IU/mL)	263.9313	51.72027

### Safety

Table 2 shows the percentage of subjects who experienced immediate local and systemic reactions as well as delayed reactions up to 28 days post vaccination. Adverse reactions for Vi-DT and Vi-PS were nearly the same (elaborated in Table 2). The most common immediate and delayed local reaction was pain whereas the most common systemic reaction was muscle pain. Delayed reactions began mostly within 24 h post vaccination. Systemic reactions recorded from 72 h to 28 days post vaccination were mostly fever which was a result of infections such as respiratory tract and gastrointestinal infections and had no causative relationship with the vaccine per se. Adverse events were of mild to moderate intensities and mostly ceased within 48 h. There were no serious adverse events.

**Table 2:** Local and systemic reactions up to 28 days

	Vi-DT (in %)	Vi-PS (in %)	P value
**Up to 30 min**			
Pain	8	6	0.579
Redness	0	0	-
Swelling	0	1	1
Induration	0	0	-
Other local reactions	0	0	-
Fever	1	0	1
Fatigue	3	1	0.673
Muscle pain	6	2	0.279
Other systemic reactions	0	0	-
**30 min to 24 h**			
Pain	12	19	0.171
Redness	1	0	1
Swelling	1	1	1
Induration	2	2	1
Other local reactions	0	0	-
Fever	2	2	1
Fatigue	4	7	0.537
Muscle pain	9	13	0.366
Other systemic reactions	1	1	1
**24 to 48 h**			
Pain	1	0	1
Redness	0	0	-
Swelling	0	1	1
Induration	0	1	1
Other local reactions	0	0	-
Fever	1	0	1
Fatigue	0	0	-
Muscle pain	0	0	-
Other systemic reactions	0	0	-
**48 to 72 h**			
Local reactions	0	0	-
Systemic reactions	0	0	-
**72 h to 28 days**			
Local reactions	0	0	-
Fever	2	4	0.327
Fatigue	0	0	-
Muscle pain	0	0	-
Other systemic reactions	1	3	0.368

P is significant if < 0.005

## Discussion

This is a phase II clinical trial of a novel Vi-DT typhoid conjugate vaccine on subjects of 12–40 years of age where Vi-PS was used as control. It was found that adverse events between Vi-DT and Vi-PS were similar which were mostly mild to moderate and ceased within 48 h. There were no serious adverse events up to 28 days. Post vaccination seroconversion as well as GMT were higher in Vi-DT compared to control.

The phase I clinical trial of this novel TCV was carried out from 2017 to 2018 and included 100 subjects divided into two age groups: 2–5 and 18–40 years old. The phase I trial concluded that this vaccine is safe with minor adverse events and immunogenic with significant seroconversion and high post vaccine GMTs. The phase I trial has been previously published [17].

With the successful results in the phase I study, the phase II trial was initiated. The phase II trial was carried out on 600 subjects divided into 3 age groups: 6 months to 23 months, 2 years to 11 years and 12 years to 40 years. The reports for the 2 former groups have already been published which showed only minor adverse events and higher seroconversion rate of Vi-DT than control was observed [18, 19]. We published them separately because Vi-DT has never been studied in children below 2 years of age, so this age group had a different control, different follow-up schedule and different solicited adverse events to look out for.

Both the phase I and II trials in the younger age groups showed that pain was the most common local reaction with muscle pain and fever as the most common systemic reactions. Similarly, local pain and muscle pain were the most common adverse reactions in this study on adults. However, up to 72 h, fever was quite rare in this age group. Fever was found after 72 h up to 28 days, but it was a result of gastrointestinal, upper airway tract infections etc. and had no causality with the vaccine. This causality was assessed by a data safety monitoring board.

A phase I clinical trial on Vi-DT TCV was also carried out in the Philippines on subjects 2 to 45 years of age with Vi-PS as control which showed similar findings as our study. Pain was the most common reaction in both this Philippines study and our present study. Post vaccination seroconversion rates and antibody GMTs were also higher in the Vi-DT group compared to Vi-PS [21].

Findings of GMT and seroconversion in this age group were consistent with our phase I trial as well as our phase II trial in the other two age groups (6–23 months and 2–11 years) which showed that GMT and seroconversion rates post vaccination were higher in Vi-DT compared to control. GMT of Vi-DT was significantly higher in Vi-DT compared to control in all our prior studies and present study with the same p value (p < 0.001) [17–19].

Another phase III trial was conducted in India on subjects 2 to 45 years using Typbar TCV as investigational vaccine and Typbar Vi-PS as control. For the 16 to 45-year-old age group, post-vaccination seroconversion rate and GMT were significantly higher in the TCV group compared to Vi-PS (p < 0.001). Most common adverse events found in the trial were fever followed by pain, however, they did not break down the adverse events based on age groups [22]. Our study is similar in terms of immunogenicity findings. Safety findings cannot be compared because the percentage of adults experiencing adverse events was unexplained.

The clinical phase II/III trial of a new TCV was carried out in India on subjects 6 months to 45 years of age. Their investigational vaccine was Vi-TT and their control was another TCV that has been licensed (Typbar). Comparing their study and our phase II trial, pain was also the most common local reaction. However, unlike our trial, theirs had fever as the most common systemic reaction. Post vaccination GMT increased significantly (p < 0.0001) [23].

Based on the results of phase I and II trials of this Vi-DT vaccine in infants, children and adults, it was demonstrated that a novel Vi-DT TCV is safe and highly immunogenic.

## Conclusion

Our phase II trial on subjects 12–40 years of age concluded that Vi-DT is safe and immunogenic in this age group.

## Data Availability

All data generated or analyzed during this study are included in this published article.
